# Effectiveness of a Skin Care Program With a Cream Containing Ceramide C and a Personalized Training for Secondary Prevention of Hand Contact Dermatitis

**DOI:** 10.1089/derm.2022.29002.flf

**Published:** 2023-03-16

**Authors:** Francesca Larese Filon, Pietro Maculan, Maria Angiola Crivellaro, Marcella Mauro

**Affiliations:** From the *Unit of Occupational Medicine, University of Trieste, Trieste, Italy.; ^†^Unit of Occupational Medicine, University of Padova, Via Giustiniani 2, 35128 Padova, Italy.

## Abstract

**Background/Objectives:**

The aim of our study was to investigate the effectiveness of personalized training on skin protection associated with the regular use of ceramide-containing cream (CC) versus other creams (OC) for improving hand contact dermatitis.

**Methods:**

We performed a double-center randomized trial that enrolled workers with hand dermatitis. All workers received personalized training. The intervention was 3 times per day application of the study emollient. The control arm used an emollient of choice without ceramide, as needed. The primary outcome was improvement in hand dermatitis at 1 and 3 months of follow-up.

**Results:**

In total, 102 patients with hand dermatitis were enrolled in this study. Improvement in dermatitis was found in 40%, 52.5%, 50%, and 63% of OC and CC, at the first and second follow-ups, respectively. The use of CC was significantly associated with an improvement in dermatitis (odds ratios 2.6; 95% confidence intervals 1.30–5.2), analyzed using generalized equation estimation during the follow-up.

**Conclusion:**

Our study demonstrated that an educational personalized intervention could improve the signs and symptoms in patients with hand dermatitis, and the use of a CC resulted in a significantly better outcome during the 3 months of follow-up.

Hand eczema is the most common work-related skin disease due to hand contact with irritant, sensitizing agents, water, and gloves.^1^ More affected workers are those exposed to “wet work” such as health care workers, hairdressers, cleaners, food operators, and so on.^2^ Hand eczema affects mostly younger workers, but prevention is possible. In fact, the maintenance of skin barrier integrity is crucial to prevent hand contact dermatitis and to avoid the recurrence of symptoms in workers with previous contact dermatitis.^3,4^ The avoidance of direct skin contact with irritants and sensitizing agents, better cleaning procedures using less aggressive detergents, and the use of moisturizing creams can play an important role in prevention.^5^

Educational interventions that teach basic rules to maintain the appropriate skin barrier have been suggested in patients with occupational skin diseases^6–10^ and can lead to a reduction in clinical signs of dermatitis^11^ as well as to improve signs of skin barrier damage.^12^

Ceramides-containing cream (CC) have been suggested as the best treatment to improve skin barrier function by increasing stratum corneum (SC) lipid levels in randomized controlled trials,^13–15^ and in atopic dermatitis patients.^16^ An ex vivo study using tape-stripped SC demonstrated that the application of a formulation containing ceramides and fatty acids resulted in a denser lateral lipid packing of the SC lipids in compromised skin.^17,18^

The aim of our study was to investigate the effectiveness of personalized training on skin protection associated with the regular use of CCs versus other creams (OC) for improving hand contact dermatitis.

## PATIENTS AND METHODS

We performed a double-center randomized trial that enrolled workers with hand dermatitis. All workers received personalized training. The intervention was 3 times per day application of the study emollient. The control arm used an emollient of choice without ceramide, as needed. The primary outcome was improvement in hand dermatitis at 1 and 3 months of follow-up.

Workers diagnosed with hand dermatitis (itching and burning of the skin with skin redness) at the Allergy Unit of the Occupational Medicine Division at the University of Trieste and University of Padua from January 2016 to May 2021 were asked to participate in the study. The inclusion criteria were contact dermatitis of the hand and agreement to participate in the study; the exclusion criteria were dermatitis that involved other body sites and chronic therapy with immunosuppressant drugs that could interfere with the outcome. Patch test was performed according to patients' compliance following our previous study protocol.^11^

All received personalized training with the aim of increasing their awareness of the importance of the adoption of preventive behaviors (regular use of emollients) and of the risk arising from skin exposure to irritants and strong detergents, drawn up on the basis of experiences described in the literature.^11,19,20^ After the training, they received a leaflet summarizing the preventive measures to apply. The hand eczema severity index (HECSI) score was used to standardize the severity of hand eczema.^21,22^

After training, patients were randomly allocated using a series of causal numbers generated by a computer into 2 groups: 1 treated with a CC and 1 treated with a traditional emollient without ceramides 3 times a day (T0). CC is characterized by the 311^®^ technology patent: 3 parts of ceramide 3, 1 part of cholesterol, and 1 part of fatty acids. Ceramide 3 seems to be the most abundant ceramide in the SC^23,24^ and, conversely, is reported to be decreased more than other ceramides in patients with atopic dermatitis and is significantly correlated with trans epidermal water loss (TEWL) impairment.^25,26^

Patients were invited to undergo a second and third clinical evaluation after 1 month (T1) and 3 months (T2). During the 3 clinical examinations, the TEWL was recorded to evaluate the skin barrier function.

All participants provided written informed consent and the procedure was approved by the ethics committee of the Friuli Venezia Giulia region (CEUR 04-2017 on July 28, 2017).

### Questionnaires Used

The questionnaire was formulated on the basis of the short version of the Nordic Occupational Skin Questionnaire^27^ with additional questions on personal and familial atopy, smoking habits, use of protective gloves, and wet time at work and out of the work.^11^ As indicators of the severity of the dermatitis, we asked about the need for dermatological examination in the past year and whether temporary sick leave for dermatitis occurred. Exposure to irritants, hand washing procedures, wet time, and use of gloves during and outside the work were assessed using the questionnaire suggested by Uter et al in 2018^28^ to investigate irritant exposure in detail. The scores achieved in the single items were summed to obtain the total irritant exposure during and outside the work.

### TEWL Measurements

TEWL measurements were performed using a VapoMeter^®^ (Defin Technologies Ltd, Kuopio, Finland), a closed-chamber type tool.^11^ Values are expressed in mg/(m^2^·h). We evaluated the volar forearm and first interdigital space as expression of areas at lower and higher exposure to irritants and/or sensitizers.^11^ Three subsequent independent samples were performed at each of these anatomic sites, and then the average was calculated. Before the measurements, each subject was invited to uncover the area of interest and to acclimatize for at least 15 minutes at a room temperature of 20–22°C and relative humidity between 40% and 60% according to guidelines and our experience.^11,29–31^ All subjects avoided the application of topical products on the measurement areas 12 hours before testing.

### Training Program

A physician analyzed the skin conditions of the patients and their exposure to irritants and sensitizing agents during and out of work using a questionnaire. Subsequently, personalized training was performed to provide information on skin anatomy, SC function, role of irritants in skin damage, penetration into the skin of irritants and sensitizing agents, role of detergents in skin damage, and use of less irritant products for cleaning procedures and emollients to restore the SC and prevent or at least reduce skin damage.^6,11^ Patients discussed how to improve their skin conditions in detail on their habits.

### Sample Size

A total of 44 subjects in each group would provide 80% power needed to detect differences between improvement of dermatitis in 60% of patients in cases and 30% in controls at the 95% confidence level. A dropout rate of 15% was predicted.

### Statistical Analysis

Data were collected using an Excel spreadsheet and processed using the STATA software, version 17.1 (Stata Corp, State College, Texas). Continuous data are expressed as median and 25°–75° percentiles and were compared using the Mann–Whitney test for independent samples and Wilcoxon signed-rank test for repeated measures in the same subject. Categorical data are expressed as numbers and percentages and compared between cases and controls using the chi-squared test. Follow-up data were investigated using generalized estimating equations, which improved the skin condition and assessed the associated factors as dependent variables. Statistical significance was set at *P* < 0.05.

## RESULTS

**Figure 1.**
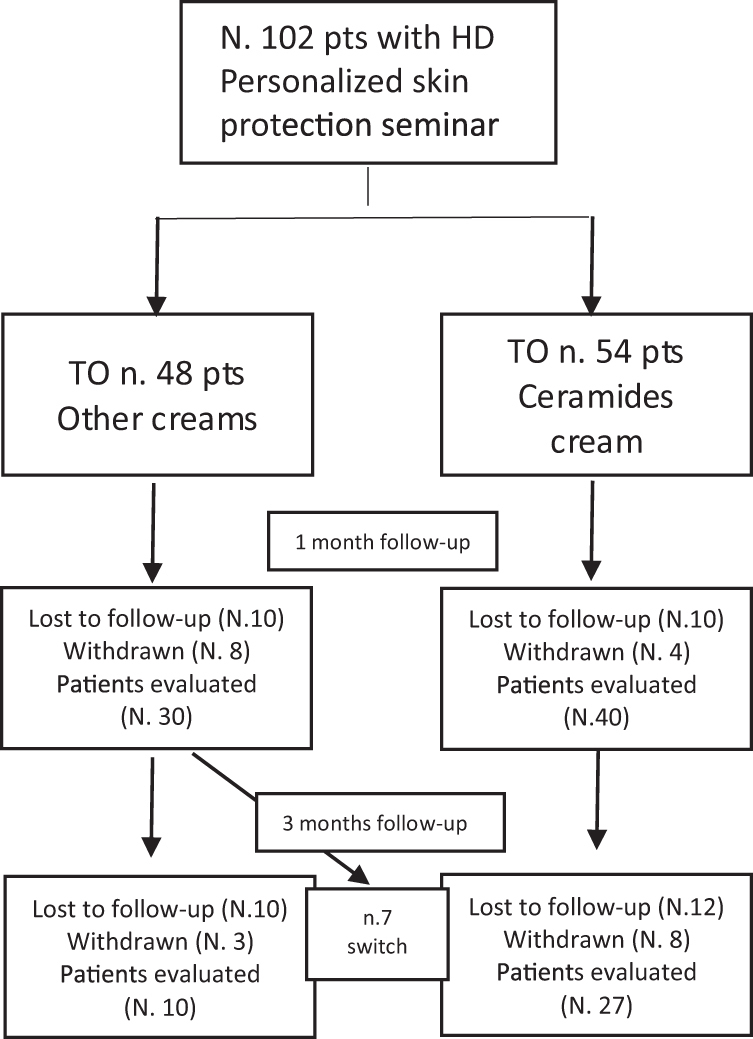
Lay-out of the study.

From January 2016 to May 2021, 102 patients with hand dermatitis of possible occupational origin agreed to participate in the study, underwent a clinical examination, and attended personalized skin protection seminars; 54 of them were randomly assigned to a group that received CC and 48 used OCs without ceramides (T0). The layout of this study is shown in Figure 1. After 1 month (T1), 40 (74%) and 30 (62.2%) patients in the CC and OC groups, respectively, participated in the first follow-up investigation. Dropped subjects were younger and had milder dermatitis (data not shown).

Seven patients in the control group were switched to the ceramide group. After 3 months, 27 and 10 patients underwent the second follow-up examination, respectively, whereas 20 and 13 patients dropped out, respectively. Dropout subjects responded to a phone interview where information on skin conditions was collected; 50% declared an improvement of the dermatitis, and for that reason, they refused the follow-up examinations.

The characteristics of subjects at recruitment are reported in Table 1, considering the group treated with CC and those treated with OC. At baseline, the demographic characteristics were similar in the considered group, and health care workers, food handlers, and mechanics were more represented by the professional group. The need to change jobs due to dermatitis was reported by 11.1% of patients receiving CC and 6.2% of workers who used OCs. Atopic dermatitis in childhood has been reported to occur in 18.5% and 14.6% of children with CC and OCs, respectively. No differences were observed in hand washings per day, dermatitis sites, or gloves-related symptoms between the 2 groups.

**Table 1.** Characteristics of the Population Studied at Baseline (*T* = 0), After 1 Month (T1), and After 3 Months (T2) in Subjects Treated with Ceramides Cream and Other Creams

	**Other Creams**			**Ceramides Cream**		
	**T0, *n* = 48**	**T1, *n* = 30**	**T2, *n* = 10**	**T0, *n* = 54**	**T1, *n* = 40**	**T2, *n* = 27**
Female, n (%)	36 (75.0)	19 (63.3)	5 (50)	32 (66.7)	26 (65)	15 (55.6)
Age median years (25°–75° percentiles)	35 (28–48)	38 (29–48)	47.5 (42–49)	36 (27–46)	41 (28–49)	39 (23–48)
Job seniority median years (25°–75° percentiles)	7 (2–17)	10 (2–22)	17 (6–24)	10 (3–21)	10 (3–20)	11 (3–23)
Occupation, n (%)						
Health care workers	27 (56.2)	18 (60)	4 (40)	24 (44.4)	16 (40)	9 (33.3)
Cleaners	1 (2.1)	0	0	1 (1.9)	1 (2.5)	1 (3.7)
Construction workers	2 (4.2)	2 (6.6)	1 (10)	3 (5.6)	2 (5)	3 (11.1)
Hairdressers	2 (4.2)	0	0	2 (3.7)	2 (5)	2 (7.4)
Mechanics	6 (12.7)	2 (6.6)	2 (20)	5 (9.2)	6 (12.5)	4 (14.8)
Food handlers	7 (14.6)	1 (3.3)	0	8 (14.8)	8 (17.5)	5 (18.5)
Others	3 (6.2)	6 (20.1)	3 (30)	11 (20.4)	6 (12.5)	3 (11.1)
Job changes due to dermatitis, n (%)	3 (6.2)	2 (6.9)	1 (10)	6 (11.1)	6 (15.0)	4 (14.8)
Atopic dermatitis in childhood, n (%)	7 (14.6)	2 (6.9)	1 (10)	10 (18.5)	8 (20)	5 (18.5)
Atopic status, n (%)	26 (54.2)	16 (55.1)	4 (40)	21 (38.9)	19 (47.5)	13 (48.1)
Rhinoconjunctivitis	15 (31.2)	9 (30.0)	2 (20)	9 (16.7)	8 (20.0)	6 (22.0)
Asthma	8 (16.8)	3 (10.0)	0	6 (11.1)	5 (12.5)	5 (18.5)
Familiar atopy, n (%)	18 (37.5)	13 (43.3)	5 (50)	20 (37.0)	14 (35.0)	8 (29.6)
Smokers, n (%)	16 (33.3)	9 (30.0)	5 (50)	13 (24.1)	11 (27.5)	7 (25.9)
Hand washings per day						
<6	14 (29.2)	7 (23.3)	3 (30.0)	11 (20.4)	9 (22.5)	7 (25.9)
6–10	13 (27.1)	7 (23.3)	1 (10.0)	12 (22.2)	11 (27.5)	8 (29.6)
11—19	12 (25.0)	7 (23.3)	3 (30.0)	13 (24.1)	11 (27.5)	6 (22.2)
>20	5 (10.4)	9 (30.0)	3 (30.0)	12 (22.2)	9 (22.5)	6 (22.2)
Dermatitis sites, n (%)						
Palm	20 (41.7)	13 (43.3)	6 (60.0)	14 (25.9)	15 (37.5)	10 (38.5)
Dorsum	21 (43.7)	8 (26.7)	2 (20.0)	22 (40.7)	20 (50.0)	14 (53.8)
Fingers	30 (62.5)	14 (46.7)	6 (60.0)	26 (48.1)	21 (52.5)	11 (42.3)
Wrist	5 (10.4)	2 (6.7)	0	8 (14.8)	7 (17.5)	3 (11.5)
Use of gloves >3 hours/day, n (%)	41 (85.4)	16 (53.3)	3 (30.0)	50 (92.6)	21 (52.5)	18 (66.7)
Glove-related symptoms, n (%)	27 (56.2)	13 (43.3)	3 (30.0)	22 (40.7)	19 (47.5)	13 (48.5)
Itching	20 (41.7)	11 (36.7)	3 (30.0)	20 (37.0)	15 (37.5)	10 (37.0)
Allergic contact dermatitis	9 (18.7)	(16.7)	1 (10.0)	10 (18.5)	7 (17.5)	5 (18.5)
Urticaria	2 (4.2)	1 (3.4)	0	1 (1.8)	1 (2.5)	2 (9.1)
Patch test done, n (%)	29 (60.4)			37 (68.5)		
Patch test negative, n (%)	13 (44.8)			9 (24.3)		
Patch test positive, n (%)	16 (55.2)			28 (75.7)		
TEWL forearm, g/m^2^/h	**9 (6–17)**	12 (7–18)	8 (4–21)	12 (8–26)^*^	13 (8–25)	**7.5 (6–12)** †
TEWL hand, g/m^2^/h	**17 (13–23)**	20 (16–25)	14 (10–20)	25 (16–39)^*^	20 (13–31)	**18 (13–30)** ‡
HECSI score	8 (3–20)	8 (4–18)	**12 (0–23)** *‡*	6 (4–17)	5.5 (2–18)	5 (2–7)
Irritant score at work	4 (2–6)	4 (1–9)	3.5 (0–5)	4 (2–7)	4.5 (2–8)	7 (3–14)
Irritant score out of work	1 (0–3)	2 (0–5)	0.5 (0–2)	1 (0–3.5)	1 (0–3.5)	3 (1–5)
Improvement, n (%)		12 (40)	5 (50)		21 (52.5)	17 (63)

^*^ *Chi-square test between ceramide creams and other creams at T0.*

† *Wilcoxon signed-rank test between the same subjects at T1 and T2 (P = 0.01).*

‡ *Wilcoxon signed-rank test between the same subjects at T1 and T3 (P < 0.02).*

*HECSI, hand eczema severity index; TEWL, trans epidermal water loss.*

*Values in bold are reported significant values.*

Patch tests were performed in 60.4% and 68.5% in OC and CC groups, respectively. Patch test resulted positive in the 55.2% and 75.7% in OC and CC groups. Nickel (n = 17), rubber additives (n = 12), and cobalt (n = 9) were the more prevalent haptens.

TEWL measured in the hands and forearms was significantly higher in the CC group, whereas no differences were observed in the HECSI score, irritant scores at work, or out of work. Improvement in dermatitis at the first follow-up was found in 40% and 52.5% of OC and CC patients, respectively, and at the second follow-up in 50% and 63% of OC and CC patients, respectively.

**Figure 2.**
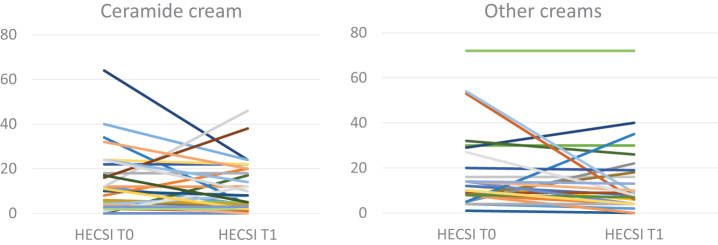
HECSI score trend from T0 to T1 in subjects treated with CC and OCs. HECSI, hand eczema severity index; CC, ceramide cream; OCs, other creams.

**Figure 3.**
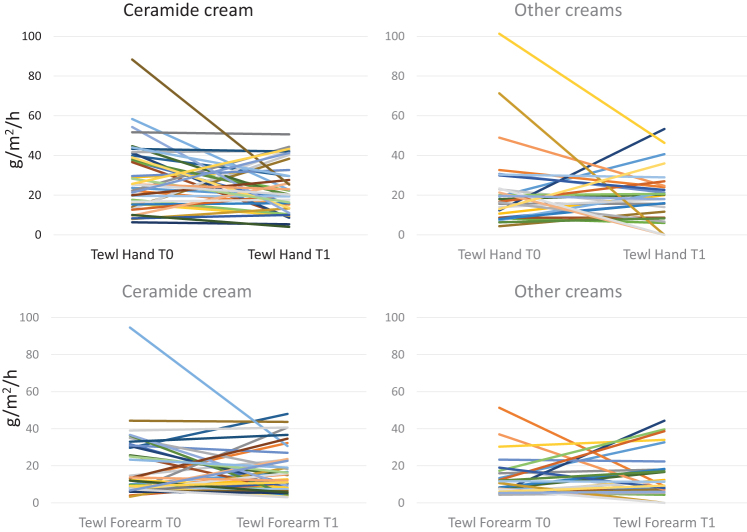
TEWL measurement at hand and forearm at T0 and T2 in subjects treated with CC and OCs. CC, ceramide cream; OCs, other creams; TEWL, trans epidermal water loss.

To better analyze the HECSI results, we reported the scores at T0 and T1 in the 2 groups (Fig. 2), showing a wide variability in the scores between subjects. Similar results were obtained for the TEWL trend in the hands and forearms of both groups (Fig. 3).

**Figure 4.**
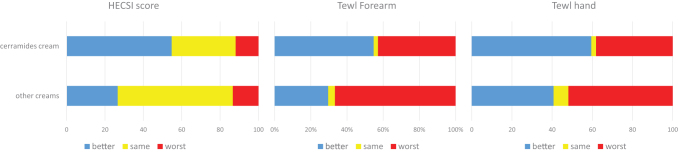
HECSI score, TEWL at hand and forearm trends (better, same, or worse) at T0 and T1 in subjects treated with CC and OCs. CC, ceramide cream; HECSI, hand eczema severity index; OCs, other creams; TEWL, trans epidermal water loss.

We analyzed the percentages of subjects that during the first follow-up obtained a better, same, and worst HECSI score and TEWL at the forearm and harm, showing that subjects treated with CC presented overall better results for the 3 considered parameters (Fig. 4). The HECSI score improved in 54.8% and 26.7% of the patients in the CC and OC groups, respectively (*P* < 0.02). TEWL measured on the hand improved in 59.5% versus 40.7% in the CC versus OC group, whereas TEWL measured on the forearm improved in 54.8% versus 29.7% in the CC versus OC group.

Factors associated with an improvement in dermatitis were evaluated during the follow-up using the generalized estimation equation, and associated factors were reported as odds ratios (ORs) and 95% confidence intervals (CIs) (Table 2); we found that the use of CC was significantly associated with an improvement in dermatitis (OR 2.6; 95% CI 1.3–5.2). Age was inversely associated with improvement (OR 0.96; 95% CI 0.92–0.97), indicating that young people presented a better outcome than older workers. For women, glove-related symptoms and the number of hand washings per day did not influence the obtained results.

**Table 2.** Factors Associated with the Improvement of Dermatitis Evaluated Using the Generalized Estimation Equations

**Factors**	**Odds Ratios (95% Confidence Intervals)**	***P***
Women	0.96 (0.43–2.15)	Ns
Age	**0.95 (0.92–0.07)**	**0.000**
Gloves-related symptoms	1.2 (0.60–2.5)	Ns
Hand washing	0.93 (0.67–1.3)	Ns
Ceramides-based creams	**2.6 (1.30–5.2)**	**0.006**

*Associations are reported as odds ratios and 95% confidence intervals.*

*Ns, not significant.*

## DISCUSSION

Our study involved 2 groups of consecutive patients who were investigated for hand dermatitis; the population was in the majority constituted by women (66.7% in both groups) due to the higher prevalence of health care workers (50%) in our population and in line with other reports on higher risk professions.^6,11,32^

Job changes due to hand dermatitis were reported in 11.1% of cases and 6.2% of controls, which could be an expression of worst-hand eczema. Moreover, cases presented a higher prevalence of atopic eczema in childhood and higher values in TEWL measured at the hand and forearm (reaching statistical significance in both cases) than controls. It is well known that atopic eczema is a predisposing factor for hand dermatitis due to the impaired skin barrier these patients have^33^; however, the prevalence of atopic eczema in Italy is lower than that reported in Nordic countries.^34^

Both groups were characterized by a high prevalence of smoking habits (>24%), and smoking is considered a risk factor for eczema, probably in relation to the inflammatory effects of smoking that cause oxidative stress and cytokine release.^35,36^ In our previous study,^11^ we demonstrated that people without hand eczema reported a significantly lower prevalence of smoking habits.

Cases and controls presented a similar distribution of hand eczema sites, similar exposure to irritants as reported by the frequency of hand washing per day, and a general irritant score at work and out of work defined according to the Uter et al questionnaire.^28^ Contact sensitization resulted a bit higher for CC compared with OC, without reaching statistical significance, and rubber additives were the more prevalent occupational haptens.

During the follow-up period, younger subjects were lost because of mild dermatitis. However, 52.5% and 40% of the subjects who participated in the first follow-up reported improvement in hand eczema in the cases and controls, respectively. These percentages increased to 63% and 50% in the case and control groups, respectively, at the second follow-up. Therefore, for both groups, the training received and the regular use of emollients were effective in reducing signs of hand eczema.

Going in detail and analyzing objective data, the HECSI score improved in higher percentages in workers treated with a CC (54.8%) than in those treated with other emollients (26.7%), and a similar trend was observed for TEWL measurements on the hand and forearm. Moreover, the median HECSI score values significantly increased in controls at the third follow-up, but only a few patients participated in the final examination, probably those with more symptoms. Notably, our patients were characterized mainly by mild dermatitis (HECSI score ≤11), for which the benefit of the interventions was higher.^12^

Educational interventions are important to teach workers to avoid exposure to irritants and to increase the use of emollient and protective creams, and many studies have reported its effectiveness in preventing hand dermatitis in hairdressers,^37^ health care workers,^38–40^ and metalworkers^41^; however, a Cochrane review^20^ on interventions to prevent occupational irritant hand dermatitis concluded that “at present there is insufficient evidence to confidently assess the effectiveness of interventions used in the primary prevention of occupational irritant hand dermatitis.”

Our previous study on secondary prevention of hand dermatitis^11^ demonstrated a significant reduction in the sign of hand eczema in 61.9% of subjects who strictly adhered to the protocol, as well as a reduction in TEWL. In this study, we obtained a similar result after 3 months, but the improvement was significantly higher in the group treated with CC, as confirmed by objective measures such as HECSI and TEWL improvement.

The statistical analysis performed using the generalized estimated equation during the follow-up confirmed the effectiveness of the treatment with CCs versus OCs (OR = 2.6; 95% CI 1.30–0.2), whereas age inversely associated with improvement, to confirm that intervention was more effective for young people, as reported by Soltanipoor et al in their cluster-randomized trial on the effectiveness of a skin care program for the prevention of contact dermatitis in health care workers.^12^ Moreover, it is known that penetration of ceramides into the skin declines with age, so it is expected that older workers presented a worse hand eczema with less recovery.^42^

Many studies have confirmed the effectiveness of CC as a treatment for impaired skin barrier function.^14,43^ Ceramides are the major lipid components (with cholesterol and free fatty acids) in the intercellular spaces of the SC that form the epidermal permeability barrier. Alterations of ceramides into the skin are associated with compromised permeability barrier functions, such as atopic dermatitis, psoriasis, and xerosis.^14^

In atopic dermatitis, the water barrier function is altered due to a dysregulation of lipid composition of SC with a reduction of ultra-long acyl chain ceramides (>C24) and an increase of long acyl chain ceramides (<C24). The barrier function can be restored with topical application of CC.^44–46^ Moreover, the use of CCs is permitted to improve atopic dermatitis in children,^17,42^ in adults with dry skin,^13,43^ and in psoriasis.^47^

In our study, the use of a CC resulted in a higher percentage of subjects with an improvement in dermatitis, with a reduction in HECSI score and a decrease in TEWL values at both sites analyzed, confirming a positive effect on the skin barrier. Analyzing our results considering *ex vivo* available data on the effectiveness of ceramide treatment demonstrated that topically applied ceramides interact with the SC lipid matrix in compromised *ex vivo* skin.^18^

The application of a formulation containing 1 or 2 ceramides and a fatty acid on regenerating the SC resulted in a denser lateral lipid packing of the SC lipids in compromised skin, and the strongest effect was observed after application of a formulation containing a single ceramide. Several other formulations consisting of ceramides, fatty acids, and cholesterol have been reported to enhance barrier repair, using TEWL as a barrier repair parameter.^48–50^

In our case, we used a cream formulated using one ceramide, ceramide 3, fatty acid, and cholesterol in a 3:1:1 ratio, which resulted in effective barrier repair in the majority of treated workers after 3 months. Nevertheless, improvement of the skin condition was also seen in the group treated only with OCs, because the use of emollients associated with better protection of the skin can be effective in improving skin condition. However, in this group at the 3 months' follow-up, we found an increase in the HECSI score, meaning that for some patients, hand dermatitis was the worst.

After the training, workers did not reduce contact with irritants and wet work, meaning that it was very difficult to change their habits during work and outside the work. Wet work, glove use, contact with detergents, and disinfectants are intrinsic factors in many occupations such as health care workers, hairdressers, and food handlers, and it is very difficult to change irritant exposure mainly at work.^12^ A better design of training would be important to improve the effectiveness of interventions.^20,51^

The results obtained in our study were mainly related to the better hydration of the skin than to a reduction in contact with irritants. This result is probably related not only to the hydrating action of the cream but also to the skin barrier-restoring action due to the 3 biomimetic balanced lipids contained in Ceramol cream.^52,53^

In a study by Sultanipoor et al,^12^ a skin care program for the prevention of contact dermatitis in health care workers was effective in both cases and controls, but with a relative improvement in HECSI significantly larger in the intervention group who received a protective cream on a regular basis during work. However, they found a decrease in natural moisturizing factors at follow-up compared with baseline, which was the opposite of what was expected.

Our study has several strengths. There are few available data in the literature that compare personalized training and CC for secondary prevention of hand dermatitis using a standardized protocol to score hand dermatitis and TEWL as markers of improvement in skin condition. The second strength is the dermatological examination of skin conditions performed during follow-up.

Moreover, our study has some limitations.

The first limitation is the high number of subjects lost to the first and second follow-up. The majority of patients presented with milder hand dermatitis and did not return for follow-up visits because they solved, at least in part, their skin problems. The second limitation is the short follow-up period; the 3 months' period was possible to register an improvement in hand dermatitis, but a longer follow-up period would permit the evaluation of the persistence of the improvement over longer time frames. A third limitation could be the higher values for TEWLS found in cases than in controls, with a possible easier positive effect due to the intervention. Another limitation could be the lack of analytical measures of compliance with the regimen (ie, quantity of creams used per day) because only personal declaration of patients was used.

## CONCLUSIONS

Our study demonstrated the effectiveness of personalized training associated with the use of emollient creams in the improvement of hand dermatitis and the more than double positive effect of a cream containing 3 parts of ceramide C, 1 part of fatty acids, and 1 part of cholesterol. Prolonged follow-up is needed to verify the long-term effects of the observed improvement.
